# HIV-1 Genetic Diversity and Drug Resistance Mutation Profiles in Donetsk, Luhansk and Zaporizhzhia Regions

**DOI:** 10.3390/v18010147

**Published:** 2026-01-22

**Authors:** Anastasiia Antonova, Anatolii Vinokurov, Daria Kustova, Andrei Pochtovyi, Daria Ogarkova, Anna Kuznetsova, Ruslan Adgamov, Elena Tsyganova, Inna Kulikova, Andrei Plutnitskii, Aleksandr Gintsburg, Vladimir Gushchin, Aleksei Mazus

**Affiliations:** 1 Federal State Budget Institution “National Research Center for Epidemiology and Microbiology Named After the Honorary Academician N.F. Gamaleya”, Ministry of Health of the Russian Federation, 123098 Moscow, Russia; 2 Department of Medical Genetics and Postgenomic Technologies, Sechenov First Moscow State Medical University, 119991 Moscow, Russia; 3 Moscow City Center for AIDS Prevention and Control, 105275 Moscow, Russia; 4 Department of Emergency Medical Assistance Organisation and Health Risk Management, Ministry of Health of the Russian Federation, 127994 Moscow, Russia; 5 Federal Medical Biophysical Center Named After A.I. Burnazyan, 123098 Moscow, Russia; 6 Department of Infectiology and Virology, Sechenov First Moscow State Medical University, 119991 Moscow, Russia; 7 Department of Virology, Lomonosov Moscow State University, 119234 Moscow, Russia

**Keywords:** HIV-1, genetic variants, genetic diversity, drug resistance mutations, EECA, DTG, RAL

## Abstract

The first major HIV outbreak in the Eastern Europe and Central Asia (EECA) region was registered. Phylogeographic analysis revealed that the main exporters of the virus were Donetsk and Lugansk, from which most migration events occurred, and the predominant genetic variant in Donetsk was subtype A. However, despite a relatively high level of understanding of HIV genetic diversity, data on resistance mutations remain limited. The aim of this study is to assess HIV genetic diversity and drug resistance in Donetsk, Luhansk and Zaporizhzhia regions. A comprehensive examination was conducted, encompassing 392 sequences covering the integrase-coding region of the HIV-1 *pol* gene. Subtyping was achieved through various programs, including COMET, the Stanford Database, BLAST and REGA. The study also involved phylogenetic analysis to clarify HIV genovariants. The profiles and levels of drug resistance were determined. The overall prevalence of drug resistance mutations to the integrase strand transfer inhibitors (INSTIs) among the studied patients was 3.6% (95% CI, 1.7–5.4%). The most commonly detected major DRMs for INSTIs were G140R (4, 28.6%) and Y143R (3, 21.4%), followed by R263K (2, 14.3%), G140RG (2, 14.3%), Y143YS (2, 14.3%), Y143YC (1, 7.1%) and Q148QR (1, 7.1%). A high-level resistance was observed for RAL—8/14 (57.1%), CAB—6/14 (42.9%) and EVG—2/14 (14.3%). The results presented are part of a further larger study and are preliminary. The results of this study suggest a moderate HIV-1 resistance situation in the Donetsk, Luhansk and Zaporizhzhia regions, but require further monitoring.

## 1. Introduction

The first major HIV outbreak in the Eastern Europe and Central Asia (EECA) region was registered among injection drug users (IDUs) in the southern regions of Ukraine (Odessa and Nikolaev), from where the subtype A virus (currently classified as sub-subtype A6) rapidly spread through drug trafficking networks across the territory of the post-Soviet countries in the 1990s [1,2,3,4,5]. Subsequently, the route of transmission shifted from parenteral to sexual, mainly due to heterosexual contact [6].

One of the factors influencing the spread of HIV infection is population migration activity. Phylogeographic analysis revealed that the main exporters of the virus were Donetsk and Lugansk, from which most migration events occurred [7], and the predominant genetic variant in Donetsk was subtype A [2].

Currently, Europe is experiencing an increase in the number of HIV infections caused by migration flows from Ukraine and the A6 sub-subtype [8]. Also, the studies indicate that approximately 69% of refugees from Ukraine received a dolutegravir (DTG)-based regimen and 22% had received an efavirenz (EFV)-based ART. The other medications employed included lopinavir/ritonavir (LPV/r) at a rate of 6% and bictegravir (BIC) at a frequency of 2% [9].

However, despite a relatively high level of understanding of HIV genetic diversity, data on resistance mutations remain limited. At the same time, the “HIV Drug Resistance—Brief Report 2024” (the World Health Organization, WHO) noted that actual levels of drug resistance to dolutegravir (DTG), a promising second-generation antiretroviral drug from the integrase strand transfer inhibitors, were higher than those observed in clinical trials. In Ukraine, in the period of 2020–2021, the overall prevalence of HIV drug resistance to DTG was 3.7%, and among individuals already receiving dolutegravir-containing antiretroviral treatment (ART) regimens, it was 6.6% [10]. Thus, there is currently a risk of the spread of drug-resistant HIV-1 variants, particularly to DTG, from Donetsk to Europe and EECA countries, including the Russian Federation.

The aim of this study is to assess HIV genetic diversity and drug resistance in Donetsk, Luhansk and Zaporizhzhia regions. This study presents preliminary results that are part of a larger scientific study.

## 2. Materials and Methods

### 2.1. Study Participants and Data Collection

Plasma samples (n = 193) and whole blood samples (n = 199) collections were used as study material. We used a consecutive sampling method. The study included patients who visited regional AIDS centers as part of their routine follow-up and registration in 2025. These centers serve as the primary point of medical care and ART distribution for all registered HIV-positive individuals in these regions. Therefore, the vast majority of the regional patient population, regardless of their gender, age and route of infection, passes through these facilities. This makes our sample representative of the population.

These biological samples were obtained from unique patients with HIV (naive and ART-treated) from three medical institutions in the Donetsk, Luhansk and Zaporizhzhia regions in 2025. The HIV sequences obtained, along with the corresponding demographic, clinical, and epidemiological information, were analyzed. These sequences covered the integrase-coding region of the HIV-1 *pol* gene.

Adherence to the Declaration of Helsinki was ensured by obtaining informed consent from all HIV-positive individuals participating in this research. To protect patient privacy, all data were anonymized and assigned unique codes.

### 2.2. HIV-1 RNA/DNA Extraction and Sequencing

Blood collection and fractionation procedures to obtain blood plasma were carried out by employees of the medical institutions in the Donetsk, Luhansk and Zaporizhzhia regions.

Commercial genotyping kits (the HiPure Viral RNA Kit (Magen Biotechnology Co., Ltd., Guangzhou, China) and the HiPure Blood DNA Mini Kit (Magen Biotechnology Co., Ltd., Guangzhou, China) were used for HIV-1 RNA extraction from plasma samples and for HIV-1 DNA extraction from whole blood, respectively.

The AmpliSens^®^ HIV-Monitor-FRT Quantitive (Central Research Institute of Epidemiology, Moscow, Russia) commercial kit was used to determine viral load. In cases when the viral load was undetectable (<1000 copies/mL), further work was carried out with proviral DNA.

In-house methods were used for Sanger-based sequencing of the HIV-1 *pol* gene regions encoding the INT (4230–5096 bp, according to the HXB2 strain, GenBank accession number K03455).

### 2.3. Determination of the HIV-1 Genetic Variants

Firstly, multiple sequence alignments were generated using the ClustalW module integrated into the AliView v.1.27 software package [11]. In case of detection of problematic regions in the resulting alignments, additional manual alignments were performed. The final alignment covered a minimum of 696 nucleotides in length (4230–5096 bp, according to the HXB2 strain, GenBank accession number K03455).

Initial identification of HIV-1 genetic variants was carried out using the following online tools: COMET HIV-1 (https://comet.lih.lu/, accessed on 26 December 2025), the HIVdb Program Sequence Analysis (available via the Stanford University website), HIV BLAST (version 2.2.30) (https://www.hiv.lanl.gov/content/sequence/BASIC_BLAST/basic_blast.html, accessed on 26 December 2025) and the REGA HIV-1 Subtyping Tool (version 3.46), following the protocol described in a previous study [12].

To validate the genotyping results, phylogenetic analysis was conducted. Reference sequences for HIV-1 genetic variants were downloaded from the Los Alamos National Laboratory HIV Sequence Database (https://www.hiv.lanl.gov/content/sequence/NEWALIGN/align.html, accessed on 26 December 2025) and incorporated into the analysis.

The phylogenetic analysis was performed by the maximum likelihood method using IQ-TREE (version 2.0.3) [13] with the following command-line arguments: iqtree-s [sequence alignment file]-m MFP-bb 1000-alrt 1000. The analysis incorporated 1000 bootstrap replicates alongside the Shimodaira–Hasegawa approximate likelihood ratio test (SH-aLRT). Clusters with SH-aLRT support values above 0.9 were considered statistically significant. The best-fit nucleotide substitution model was degermed automatically

The resulting phylogenetic tree was visualized and annotated using iTOL (Interactive Tree of Life) software (version 7) [14].

### 2.4. Analysis of Drug Resistance Mutations

Drug resistance mutation analysis (determination of the integrase strand transfer inhibitors (INSTIs) was performed using the Sierra algorithm implemented in the HIVdb Program: Mutations Analysis Tool version 9.7 (Stanford University HIV Drug Resistance Database; https://hivdb.stanford.edu/hivdb/by-sequences/, accessed on 26 December 2025).

The Calibrated Population Resistance Tool (CPR) was used to perform drug resistance analysis among patients with HIV not on ART (PrimDR).

The Stanford HIV Drug Resistance Database (including the HIVdb Program, version 9.8, and the Calibrated Population Resistance Tool (CPR)) was used to characterize and interpret HIV drug resistance (DR) levels and drug-resistance mutations (DRMs), including surveillance drug-resistance mutations (SDRMs) [15].

DR levels were categorized into five tiers according to the Stanford penalty score: susceptible (score 0–9); potential low-level resistance (score 10–14); low-level resistance (score 15–29); intermediate resistance (score 30–59); and high-level resistance (score ≥ 60). This classification was applied for the following INSTIs: bictegravir (BIC), cabotegravir (CAB), dolutegravir (DTG), elvitegravir (EVG) and raltegravir (RAL). Sequences with a Stanford penalty score of 15 or higher were considered resistant to these drugs.

### 2.5. Statistical Analysis

The analysis was carried out using the R programming language (R version 4.2.2, RStudio version 2023.03.0 + 386, Inc. Software, Boston, MA, USA), as well as STATISTICA software (version 6.0, StatSoft, Tulsa, OK, USA).

Categorical data evaluated in the study were presented as proportions and frequencies and their comparisons were carried out using the chi-square test (χ^2^); if unstable, Yates’ correction or Fisher’s two-tailed exact test was applied. Differences were considered statistically significant at *p*-value < 0.05. Data visualization was performed using the R programming language.

## 3. Results

### 3.1. Profile of the Study Cohort

A total of 392 unique HIV-1 sequences were analyzed, obtained from patients with HIV in the Donetsk, Luhansk and Zaporizhzhia regions in 2025. Of these, 352 patients were receiving ART at the time of sampling, while 5 patients were not receiving ART, and information about the ART status for 35 other patients was not available. The most commonly used ART regimens in the study cohort of patients were TDF/3TC/DTG, TDF/3TC/EFV, TDF/FTC/ESV. The study cohort’s average duration time on ART was 101 (from 59 to 141) months, equivalent to 8 (from 4 to 11) years.

The median age of the participants (age data was available for 372 patients) was 43 years, ranging from 2 to 68 (Q1 = 36, Q3 = 49 years). Among the cohort, 204 (52.2%) individuals were male, and 187 (47.8%) individuals were female (gender data was available for 391 patients).

The main risk factor was sexual contact (265, 67.6%), followed by intravenous drug use (78, 19.9%). In the structure of sexual transmission, the heterosexual contact were most common (98.9%), followed by homosexual contact (0.7%) and unspecified (0.4%).

Data on the median (IQR) CD4 cell counts and median (IQR) HIV RNA for treated and untreated patients are presented in Table 1.

### 3.2. Phylogenetic Analysis

**Figure 1:**
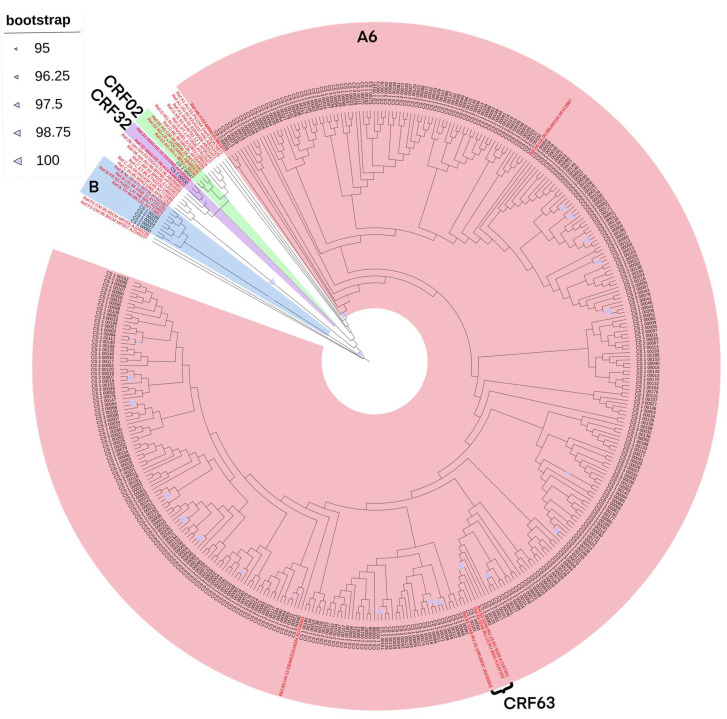
The results of phylogenetic analysis of the studied nucleotide sequences (n = 392). The reference nucleotide sequences are marked in red. The differently colored areas mark HIV-1 clusters depending on their genetic variant: pink marks the HIV-1 sub-subtype A6 cluster, blue marks B, light green marks CRF02, and purple marks CRF32.

The combined refined results of the initial identification of HIV-1 genetic variants and phylogenetic analysis showed the following ratios: A6—382 of sequences (97.45%), B—5 (1.28%), CRF63_02A6—3 (0.77%), CRF02_AG—1 (0.25%) and CRF32_06A6—1 (0.25%) of sequence (Figure 1).

### 3.3. Analysis of Drug Resistance Mutations

To assess the prevalence of key DRMs, we analyzed the INT sequences (n = 392) targeting resistance to INSTIs.

In four patients without a history of ART, no resistance mutations were detected. The overall prevalence of drug resistance mutations to the integrase strand transfer inhibitors (INSTIs) among the studied patients was 3.6% (95% CI, 1.7–5.4%) (Table 2).

The most commonly detected major DRMs for INSTIs were G140R (4, 28.6%) and Y143R (3, 21.4%), followed by R263K (2, 14.3%), G140RG (2, 14.3%), Y143YS (2, 14.3%), Y143YC (1, 7.1%) and Q148QR (1, 7.1%). Considering the different lengths of the sequences, it is possible that the frequency of mutation R263K may even be higher. One patient exhibited a combination of these mutations: G140R + R263K (major) and G163R (accessory); a further four patients had both major and accessory mutations: for two patients—Y143R + T97A, and for two patients—G140R + G163R (Table 2).

A high-level resistance to INSTIs was observed for RAL—8/14 (57.1%), CAB—6/14 (42.9%), and EVG—2/14 (14.3%). Intermediate resistance was observed to vary across all the drugs presented: EVG—8/14 (57.1%), RAL—5/14 (35.7%), BIC and DTG—3/14 (21.4%) for each drug and CAB—2/14 (14.3%) (Table 2).

Among other mutations, the most common was the L74I polymorphism, which was presented in 370 patients (94.6%, 95% CI, 92.1–96.7%).

## 4. Discussion

Overall, the results obtained reflect the current trends in HIV infection in the EECA region, within the context of demographic and epidemiological indicators [16,17]. It is noteworthy that approximately 19% of HIV transmission cases were among IDUs, which aligns with the overall results for Russia. This indicates a definite shift in transmission routes in the region, with HIV infection spreading predominantly through heterosexual contact.

In terms of HIV-1 genetic diversity, the region maintains the historically justified absolute dominance of HIV-1 sub-subtype A6 [1,2,3,4,5]. However, even within this context, the study cohort exhibits a high genetic diversity of the virus, potentially resulting from active migration processes. Additionally, the recombinant form CRF32_06A6 was identified in the study area for the first time. This recombinant form was initially identified and described in Estonia in the mid-2000s as the dominant genetic variant among IDUs [18]. According to clinical and epidemiological data, the patient with this recombinant form of HIV-1 was infected through heterosexual contact. This discovery necessitates further monitoring due to the potential for continued transmission and spread of CRF32_06A6 in the region.

Given the significant genetic fragment of the A6 genovariant the integrase-coding region of the HIV-1 *pol* gene within CRF32_06A6, it is possible that this recombinant form also carries the L74I polymorphism. This polymorphism has been shown to significantly increase the risk of virological failure when using long-acting injectable antiretroviral drugs, CAB/RPV, which are commonly used in Europe and are now included in clinical guidelines for treatment in Russia since 2024 [19,20].

Despite the low percentage (3.6%) of patients with major drug resistance mutations to INSTIs, the results obtained warrant attention and further monitoring. Notably, the most common mutation in the study cohort was G140R, a rare non-polymorphic mutation that reduces the susceptibility to CAB [21]. It can be assumed that this mutation is locally distributed among certain groups. Additionally, a specific mutational background may be required for G140R to reduce the susceptibility to CAB, as three out of four patients (75%) with this mutation also had additional major (R263K) and accessory (G163R) mutations, which may also potentially reduce the virus’s sensitivity to other antiretroviral drugs: EVG, DTG, BIC, and CAB [22,23].

The second most common mutation was Y143R, which reduces the susceptibility to RAL by approximately 20-fold. In combination with T97A, which was observed in 75% of patients with the Y143R mutation, it reduces the susceptibility by 100-fold [24,25,26,27]. It is worth noting that among patients with major DRMs, only one patient had a history of RAL use. This may indicate cases of transmitted HIV-1 drug resistance in the region. Similarly, among patients with intermediate-level resistance to DTG, none had a history of using DTG.

## 5. Conclusions

The results presented in this study are part of a further larger research project and should be considered preliminary. The results of this study indicated a moderate HIV-1 resistance situation in the Donetsk, Luhansk and Zaporizhzhia regions. However, considering several limitations of the study, including the lack of a representative sample of treatment-naive patients, further expansion of this research is necessary. Therefore, ongoing monitoring of HIV infections in these areas is essential.

## Figures and Tables

**Table 1:** Data on the median (IQR) CD4 cell counts and median (IQR) HIV RNA for the study patients.

	Untreated		Treated	
	n	Median [Q1–Q3]	n	Median [Q1–Q3]
CD4 cell counts, cells/μL	5	337 [151–602]	317	536 [344–760]
HIV RNA, copies/mL	5	13,000 [2400–19,140]	328	40 [0–40]

**Table 2:** The results of the resistance analysis of the HIV samples studied.

Name	Genetic Variant	INSTI Major	INSTI Accessory	BIC	CAB	DTG	EVG	RAL
CS_1_00003	A6	Y143YS		potential low-level	low-level	potential low-level	low-level	high-level
CS_1_00062	A6	G140R, R263K	G163R	intermediate	high-level	intermediate	high-level	high-level
CS_1_00084	A6	Y143YC		potential low-level	low-level	potential low-level	low-level	high-level
CS_1_00107	A6	R263K		intermediate	intermediate	intermediate	intermediate	low-level
CS_1_00108	A6	G140RG		potential low-level	high-level	potential low-level	intermediate	intermediate
CS_1_00127	A6	G140R		potential low-level	high-level	potential low-level	intermediate	intermediate
CS_1_00133	A6	Q148QR		intermediate	intermediate	intermediate	high-level	high-level
CS_1_00155	A6	Y143YS		potential low-level	low-level	potential low-level	low-level	high-level
CS_1_00202	A6	Y143R	T97A	potential low-level	low-level	potential low-level	intermediate	high-level
CS_2_00025	A6	Y143R	T97A	potential low-level	low-level	potential low-level	intermediate	high-level
CS_2_00073	A6	G140R	G163R	potential low-level	high-level	potential low-level	intermediate	intermediate
CS_3_00014	A6	G140R	G163R	potential low-level	high-level	potential low-level	intermediate	intermediate
CS_3_00031	A6	G140RG		potential low-level	high-level	potential low-level	intermediate	intermediate
CS_3_00042	A6	Y143R		potential low-level	low-level	potential low-level	low-level	high-level

Note: The table presents only those samples in which major mutations of resistance to INSTIs have been detected.

## Data Availability

All nucleotide sequences analyzed in the study were deposited in the Los Alamos National Laboratory International Database, accession numbers: PX796826-PX797217.
